# Intravenous tirofiban following successful reperfusion in intracranial large artery atherosclerotic stroke: A secondary analysis of a randomized clinical trial

**DOI:** 10.1002/acn3.51891

**Published:** 2023-08-30

**Authors:** Jiacheng Huang, Weilin Kong, Chang Liu, Jiaxing Song, Jie Yang, Chengsong Yue, Linyu Li, Jinrong Hu, Yan Tian, Zhouzhou Peng, Changwei Guo, Dahong Yang, Xiang Liu, Jian Miao, Xiao Zhang, Fengli Li, Jeffrey L. Saver, Wenjie Zi

**Affiliations:** ^1^ Department of Neurology Xinqiao Hospital and The Second Affiliated Hospital Army Medical University (Third Military Medical University) Chongqing 400037 China; ^2^ Department of Neurology The Second Affiliated Hospital of Chongqing Medical University 74 Linjiang Road, Yuzhong District Chongqing 400010 China; ^3^ Department of Neurology Xianyang Hospital of Yan'an University No. 38, Middle Section of Wenlin Road Xianyang 712000 China; ^4^ Department of Neurology The Affiliated Hospital of Northwest University Xi'an No.3 Hospital Xi'an 710000 China; ^5^ Department of Neurology and Comprehensive Stroke Center David Geffen School of Medicine University of California Los Angeles California 90095 USA

## Abstract

**Objective:**

This study aimed to investigate whether treatment with adjunct intravenous tirofiban is associated with improved outcomes following successful reperfusion in patients with intracranial atherosclerotic stroke.

**Methods:**

Patients with intracranial large artery atherosclerotic (LAA) stroke and an expanded Treatment in Cerebral Ischemia angiographic score of 2b50 to 3 from the Effect of Intravenous Tirofiban versus Placebo Before Endovascular Thrombectomy on Functional Outcomes in Large Vessel Occlusion Stroke (RESCUE BT) trial were included. The primary outcome was the difference in proportion of independent functional outcome (modified Rankin score of 0–2 at 90 days). Safety outcomes included the rates of symptomatic intracranial hemorrhage (sICH) and 90‐day mortality.

**Results:**

Among the 382 patients with intracranial LAA stroke and successful reperfusion, 175 patients (45.8%) were treated with intravenous tirofiban and 207 (54.2%) with placebo. The proportion of patients with independent functional outcome at 90 days was 54.3% (95 out of 175) with tirofiban and 44.0% (91 out of 207) with placebo (adjusted odds ratio [aOR], 1.58; 95% CI, 1.02–2.44; *p* = 0.04). Intravenous tirofiban was not significantly associated with an increased risk of sICH (12/175 [6.9%] vs. 11/207 [5.3%]; aOR, 1.41; 95% CI, 0.59–3.34; *p* = 0.44) or 90‐day mortality (21/175 [12.0%] vs. 34/207 [16.4%]; aOR, 0.71; 95% CI, 0.38–1.31; *p* = 0.27).

**Interpretation:**

Among patients with acute intracranial LAA stroke and successful reperfusion following endovascular thrombectomy, adjunct intravenous tirofiban was associated with a higher rate of independent functional outcome, without higher rates of sICH or mortality. Confirmatory randomized trials in these patients are desirable.

## Introduction

Endovascular thrombectomy (EVT) is the standard treatment to improve functional outcomes in acute ischemic stroke patients with large vessel occlusion.^1^, ^2^ However, despite advances in thrombectomy devices and optimization of treatment workflow processes, outcomes remain suboptimal. Notably, even when EVT yields successful reperfusion (expanded Thrombolysis In Cerebral Infarction [eTICI] scale 2b50‐3) acutely, only one half of patients achieve functional independence at 90 days.^3^, ^4^ Part of this incomplete recovery reflects infarction that accrued prior to intervention. But another important mechanism may be post‐procedure infarct progression in regions with insufficient macrocirculatory and microcirculatory reperfusion despite successful recanalization of the target large vessel occlusions at the end of procedure.^5^ A recent randomized trial showed that adjunct intra‐arterial alteplase infused after achievement of successful reperfusion with EVT improved neurological outcomes at 90 days in patients with acute ischemic stroke, suggesting that functional outcome can be improved by facilitating macrocirculatory and microcirculatory reperfusion.^6^

Among patients with successful reperfusion with EVT, residual blood flow impairment is of two types: macrocirculatory—angiographically visible occlusions in more distal arteries; and microcirculatory—no reflow at the arteriolar–capillary level despite absence of any angiographically visible occlusion. For example, in patients with eTICI 3 outcome, no visible occlusions are present but hypoperfusion throughout the field may occur due to microcirculatory obstructions.

Intracranial large artery atherosclerotic (LAA) stroke is one of the most common etiologies of large artery occlusion, especially in Asian patients.^7^, ^8^ Compared to cardiac embolism, LAA predicts a lower chance of successful reperfusion and worse functional outcomes after EVT.^9^ Recently, the Endovascular Treatment With versus Without Tirofiban for Stroke Patients with Large Vessel Occlusion (RESCUE BT) trial showed that administer intravenous tirofiban before EVT does not improve functional outcomes in patients with acute large vessel occlusion stroke.^10^ However, there was a hint of benefit with tirofiban in patients with LAA stroke, although there were no significant interactions among various subgroups. In the post hoc study, we evaluate trial findings regarding the association of adjunct intravenous tirofiban with functional outcome specifically in intracranial LAA patients in whom EVT yielded successful reperfusion.

## Methods

### Study participants

The RESCUE BT trial was a multicenter, randomized, double blinded clinical trial conducted at 55 hospitals in China from October 2018 to October 2021. Details of trial design and topline results have been described previously.^10^, ^11^ Briefly, patients with acute ischemic stroke attributed to large vessel occlusion within 24 h of time last known well were enrolled. Inclusion criteria were: (1) age ≥18 years old; (2) National Institutes of Health Stroke Scale (NIHSS) score ≤30; (3) Alberta Stroke Program Early CT Score (ASPECTS) score ≥6； (4) occlusion of the intracranial internal carotid artery, the first or second segment of the middle cerebral artery confirmed by computed tomography angiography, magnetic resonance angiography, or digital subtraction angiography; (5) without intravenous thrombolysis due to contraindication or no consent for thrombolysis given. The study protocol was approved by the ethics committees of the Xinqiao Hospital, Army medical university and all participating centers. Written informed consent was obtained from all the patients or their legally authorized representatives.

For the current post hoc study, additional inclusion criteria were, (6) diagnosis of intracranial large artery atherosclerosis as the mechanism of the index ischemic stroke, and (7) achievement of successful reperfusion at the end of the mechanical thrombectomy procedure. Successful reperfusion was defined as an eTICI grade of 2b50‐3, including 2b50 (substantial reperfusion, 50–89%), 2c (excellent reperfusion, 90–99%), and 3 (completely reperfusion, 100%).^12^ In the RESCUE BT trial, stroke mechanism was defined based on the criteria of the Trial of Org 10172 in Acute Stroke Treatment (TOAST) classification.^13^ Intracranial LAA‐related stroke was considered present when there was obvious (50–99%) residual focal stenosis at the occlusion site after mechanical thrombectomy, accompanied by 1 or more high risk factors for atherosclerosis, such as advanced age, history of smoking, diabetes, hypertension, hyperlipidemia, etc. Some of the information on risk factors may not be available at the time of the procedure, therefore, the physician can apply the clinical and imaging findings when first assessing the patient and then consider the results of other diagnostic tests later.

### Study treatments and interventions

The study drug was administered intravenously within 5 min after randomization. Eligible participants in the tirofiban group received intravenous bolus followed by continuous infusion of tirofiban (10 μg/kg bolus and then 0.15 μg/kg/min maintenance for up to 24 h), participants in the placebo group received saline. A rapid EVT was initiated. It was permitted to use rescue drug when reocclusion of the target artery after EVT was observed in the angiographic suite. The rescue drug was saline placebo in the tirofiban group and tirofiban in the placebo group. Patients who achieved successful reperfusion after use of rescue drug were included in this analysis in addition to those not requiring rescue drug.

### Outcomes measures

This analysis used the same primary and all applicable secondary efficacy analyses used for the parent trial. The primary efficacy outcome measure was the proportion of patients functionally independent (modified Rankin Scale [mRS] score of 0 to 2) at 90 days. The mRS score is an ordered scale ranging from 0 (no symptoms) to 6 (death).

Secondary efficacy outcomes included a shift analysis of the mRS score at 90 days; the proportion of patients disability‐free (mRS score 0 to 1); the change of the NIHSS score from baseline to 24 h and 5–7 days (or at discharge if earlier); the score of the European Quality of Life 5‐Dimension 5‐level scale (EQ‐5D‐5L; a higher score indicates a better quality of life) at 90 days. Safety outcomes included incidence of symptomatic intracerebral hemorrhage (sICH) according to Heidelberg Bleeding Classification,^14^ mortality within 90 days, and serious adverse events.

### Statistical analysis

Continuous variables were reported as medians (interquartile ranges) and categorical variables as numbers (percentages). Baseline characteristics between patients with and without intravenous tirofiban were compared by using the Wilcoxon rank sum test or chi‐squared test as appropriate. The main efficacy outcome was estimated using a multivariable logistic regression model adjusted for age, baseline NIHSS score, baseline ASPECTS, occlusion site, time from last known well to randomization. In a sensitivity analysis, the estimated odds ratio of effect of intravenous tirofiban on independent functional outcome was performed between the tirofiban group and the placebo group only among patients who did not receive rescue drug.

Among secondary clinical outcomes, the improvement in mRS score at 90 days was estimated by ordinal logistic regression model adjusted for the same variables above. The odds ratios for early neurological changes (the change of the NIHSS score from baseline to 24 h and 5–7 days) and quality of life at 90 days were analyzed using multivariable linear regression model. The risk ratio for safety outcomes was estimated by Poisson regression model. The unadjusted and adjusted value were presented with 95% confidence intervals to indicate statistical precision.

Subgroup analyses were performed evaluating potential heterogeneity for efficacy effect among patients with substantial reperfusion (eTICI 2b50), excellent reperfusion (eTICI 2c), and complete reperfusion (eTICI 3), using forest plot analysis. Day 90 functional outcomes analyzed included the primary outcome (mRS 0–2), the prespecified secondary mRS outcomes (mRS 0–1). Heterogeneity was assessed by interaction *p* value and *I* ^2^, which describes the percentage of variability in the estimates that is due to heterogeneity rather than chance. We considered evidence of heterogeneity to be present if the *I* ^2^ statistic was greater than 50%.

Additional subgroup analyses were performed for the primary mRS 0–2 outcome stratified by the same variables as in the overall trial analysis: age, sex, baseline NIHSS score, baseline ASPECTS, occlusion site, time from last known well to randomization. All analyses were performed using SPSS version 23 (IBM Corp.) and Review Manager 5.3. For main effect analyses, *p* ≤ 0.05 (two‐sided) was considered statistically significant. For subgroup analyses, *p* ≤ 0.10 (two‐sided) was considered statistically significant.^15^

## Results

### Baseline characteristics

**Figure 1:**
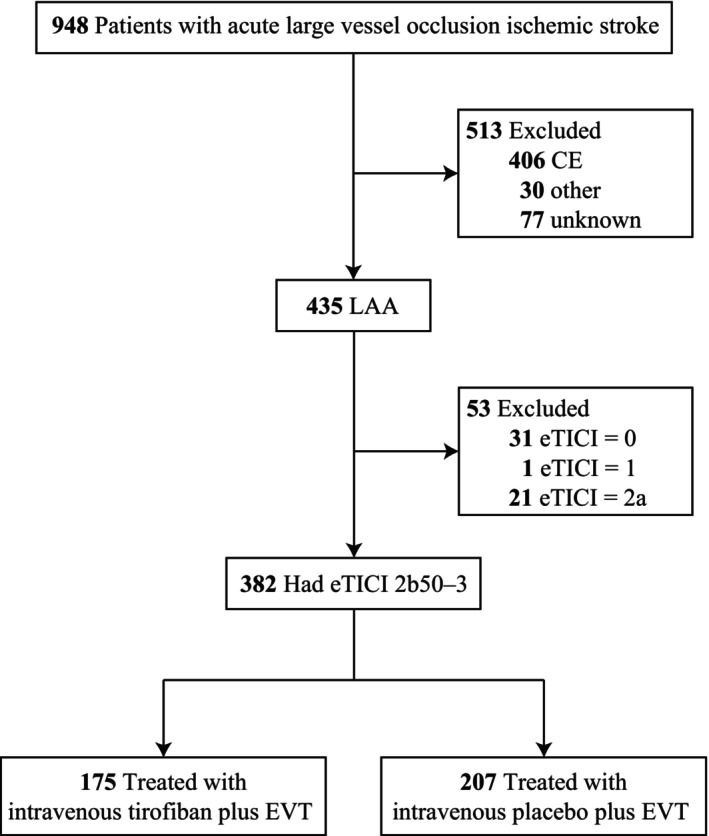
Flowchart.

Of 948 enrolled patients in the RESCUE BT study, 435 had intracranial LAA etiology, 197 in the intravenous tirofiban group, among whom 175 (88.8%) had successful reperfusion, and 238 in the placebo group, among whom 207 (87.0%) had successful reperfusion (eTICI 2b50‐3) (Fig. 1). Baseline characteristics for these 382 patients, combined and separately by treatment group, were shown in Table 1. Overall, age was median 65 (interquartile [IQR] 55–72) years, NIHSS 14 (IQR 10–18), and ASPECTS 8 (IQR 7–9). Patients features at entry were generally well balanced between the two groups, except that the tirofiban group had mildly more patients with M1 middle cerebral artery segment occlusion (77.7% vs. 68.6%) and fewer patients with M2 middle cerebral artery segment occlusion (6.9% vs. 15.0%).

**Table 1:** Baseline characteristics and workflow measures.

Characteristics	All (*N* = 382)	Tirofiban (*N* = 175)	Placebo (*N* = 207)	*p* value
Age, median (IQR), years	65 (55–72)	65 (55–71)	64 (56–73)	0.83
Sex, no./total no. (%)				0.18
Female	105 (27.5)	54 (30.9)	51 (24.6)	
Male	277 (72.5)	121 (69.1)	156 (75.4)	
Medical history, no./total no. (%)				
Hypertension	245 (64.1)	108 (61.7)	137 (66.2)	0.36
Hyperlipidemia	77 (20.2)	40 (22.9)	37 (17.9)	0.23
Artiral fibrillation	27 (7.1)	14 (8.0)	13 (6.3)	0.51
Coronary heart disease	62 (16.2)	29 (16.6)	33 (15.9)	0.87
Diabetes mellitus	103 (27.0)	47 (26.9)	56 (27.1)	0.97
Ischemic stroke	59 (15.4)	23 (13.1)	36 (17.4)	0.25
Smoking	140 (36.6)	59 (33.7)	81 (39.1)	0.27
Prestroke modified Rankin scale score ≥2*	7 (1.8)	5 (2.9)	2 (1.0)	0.25
Prestroke antithrombotic therapy, no./total no. (%)				
Antiplatelet therapy	36 (9.4)	18 (10.3)	18 (8.7)	0.60
Anticoagulant*	6 (1.6)	3 (1.7)	3 (1.4)	1.00
ASPECTS score, median (IQR)	8 (7–9)	8 (7–9)	8 (7–9)	0.33
Occlusion site, no./total no. (%)				0.04
Intracranial internal carotid artery	61 (16.0)	27 (15.4)	34 (16.4)	
M1 middle cerebral artery segment	278 (72.8)	136 (77.7)	142 (68.6)	
M2 middle cerebral artery segment	43 (11.3)	12 (6.9)	31 (15.0)	
Clinical examination at arrival, median (IQR)				
NIHSS score	14 (10–18)	14 (10–18)	14 (10–19)	0.99
Systolic blood pressure, mm Hg	148 (132–165)	149 (135–169)	147 (132–162)	0.27
Diastolic blood pressure, mm Hg	86 (77–96)	85 (77–94)	86 (76–97)	0.75
Glucose level, mmol/liter	6.97 (5.7–9.3)	7.00 (5.77–9.02)	6.95 (5.68–9.63)	0.89
Intravenous thrombolysis*	10 (2.6)	6 (3.4)	4 (1.9)	0.52
Workflow times, median (IQR), min				
Last known well to hospital arrival	340 (189–587)	365 (183–570)	335 (200–590)	0.98
Last known well to randomization	480 (328–719)	502 (327–708)	476 (330–731)	0.68
Last known well to arterial puncture	485 (309–726)	494 (310–705)	475 (305–745)	0.85
Arterial puncture to reperfusion	82 (50–128)	80 (47–120)	85 (52–131)	0.38
Balloon angioplasty, no./total no. (%)	206 (53.9)	89 (50.9)	117 (56.5)	0.27
Stenting, no./total no. (%)	108 (28.3)	54 (30.9)	54 (26.1)	0.30

ASPECTS, the Alberta Stroke Program Early Computed Tomography Score; IQR, interquartile range; NIHSS, National Institutes of Health Stroke Scale.

* *p* values were calculated using Fisher exact tests.

### Primary efficacy outcome

**Figure 2:**
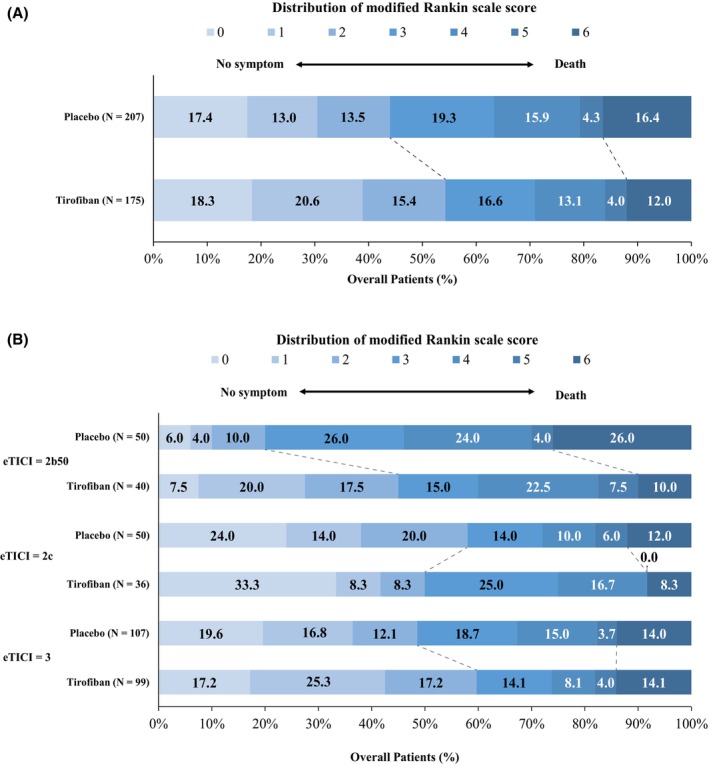
Distribution of the Modified Rankin Scale score at 90 days.

Treatment with intravenous tirofiban was associated with independent functional outcome (mRS 0 to 2) at 90 days in 54.3% (95 out of 175) patients in the tirofiban group and 44.0% (91 out of 207) patients (44.0%) in the placebo group (adjusted odds ratio [aOR], 1.58; 95% CI, 1.02–2.44; *p* = 0.04) (Table 2 and Fig. 2). Similarly, the sensitivity analysis, confined to patients with successful reperfusion without use of rescue therapy, showed that the proportion of patients achieving functional independence in the tirofiban group was significantly higher than that of the placebo group (95 out of 175 [54.3%] vs. 67 out of 156 [42.9%]; aOR, 1.60; 95% CI, 1.02–2.52; *p* = 0.04) (data not shown). The proportion of independent functional outcome showed no significant difference between the treatment groups in patients with unsuccessful reperfusion.

**Table 2:** Efficacy and safety outcomes.

Outcomes	All (*N* = 382)	Tirofiban (*N* = 175)	Placebo (*N* = 207)	Adjusted OR (95% CI)^1^	*p* value
Primary efficacy outcome					
Modified Rankin Scale score of 0–2 at 90 days, no./total no. (%)	186 (48.7)	95 (54.3)	91 (44.0)	1.58 (1.02–2.44)	0.04
Secondary efficacy outcome					
Modified Rankin Scale score at 90 days, median (IQR)^2^	3 (1–4)	2 (1–4)	3 (1–4)	0.72 (0.50–1.04)	0.08
Modified Rankin Scale score of 0–1 at 90 days, no./total no. (%)	133 (34.8)	69 (39.4)	64 (30.9)	1.52 (0.96–2.43)	0.07
Modified Rankin Scale score of 0–3 at 90 days, no./total no. (%)	255 (66.8)	124 (70.9)	131 (63.3)	1.47 (0.92–2.37)	0.11
NIHSS score changes, median (IQR)					
Change from baseline to 24 hours*	−4 (−5–2)	−1 (−5–2)	−2 (−5–3)	0.32 (−1.29–1.94)	0.69
Change from baseline to 5–7 days or early discharge*	−4 (−8–1)	−4 (−8–0)	−4 (−9–1)	−0.60 (−2.69–1.49)	0.57
EQ‐5D‐5L score at 90 days, median (IQR)*	0.71 (0.22–0.96)	0.78 (0.27–0.96)	0.68 (0.12–0.96)	0.07 (0.00–0.14)	0.04
Substantial reperfusion eTICI score, no./total no. (%)^2^				1.12 (0.76–1.67)	0.56
2b	90 (23.6)	40 (22.9)	50 (24.2)		
2c	86 (22.5)	36 (20.6)	50 (24.2)		
3	206 (53.9)	99 (56.6)	107 (51.7)		
Rescue drug use, no./total no. (%)	78 (20.4)	27 (15.4)	51 (24.6)	0.51 (0.30–0.87)	0.01
Primary safety outcomes					
Symptomatic intracranial hemorrhage	23 (6.0)	12 (6.9)	11 (5.3)	1.41 (0.59–3.34)	0.44
Any radiologic intracranial hemorrhage	90 (23.6)	51 (29.1)	39 (18.8)	1.92 (1.18–3.12)	0.009
Mortality at 90 days	55 (14.4)	21 (12.0)	34 (16.4)	0.71 (0.38–1.31)	0.27

CI, confidence interval; EQ‐5D‐5L, European Quality of Life 5‐Dimension 5‐level scale; eTICI, expanded Thrombolysis In Cerebral Infarction grade; IQR, interquartile range; NA, not applicable; NIHSS, National Institutes of Health Stroke Scale; OR, odds ratio.

^1^ Values were adjusted for age, baseline NIHSS score, baseline ASPECTS, occlusion site, and time from last known well to randomization.

^2^ Values were calculated using ordinal logistic model.

* Values were calculated using linear regression model.

### Secondary efficacy outcomes

The secondary clinical efficacy outcomes were shown in Table 2. Intravenous tirofiban was associated with improved quality of life (aOR, 0.07; 95% CI; 0.00–0.14; *p* = 0.04). The proportion of using rescue drug was significantly lower in the tirofiban group than placebo group (27 out of 175 [15.4%] vs. 51 out of 207 [24.6%]; aOR; 0.51; 95% CI; 0.30–0.87; *p* = 0.01).

### Safety outcomes

At 90 days, no significant difference was observed between the two groups in the incidence of sICH, 12 out of 175 (6.9%) versus 11 out of 207 (5.3%) (aOR, 1.41; 95% CI, 0.59–3.34; *p* = 0.44), or the incidence of death, 21 out of 175 (12.0%) versus 34 out of 207 (16.4%) (aOR, 0.71; 95% CI, 0.38–1.31; *p* = 0.27), respectively. Nevertheless, the proportion of any radiologically visible intracranial hemorrhage was significantly higher in the tirofiban group compared with the placebo group, 51 out of 175 (29.1%) versus 39 out of 207 (18.8%) (aOR, 1.92; 95% CI, 1.18–3.12; *p* = 0.009). Other safety outcomes were similar between the two groups in both the overall cohort (Table 3). In patients due to non‐LAA stroke, higher proportion of sICH and 90‐day mortality were observed in the tirofiban group compared with the placebo group (Table S1 in the Supplement).

**Table 3:** Safety outcomes of the cohort.

Additional safety outcomes	All (*N* = 382)	Tirofiban (*N* = 175)	Placebo (*N* = 207)	Risk ratio (95% CI)	*p* value
Acute respiratory failure	52 (13.6)	23 (13.1)	29 (14.0)	0.94 (0.56–1.56)	0.81
Acute heart failure	37 (9.7)	16 (9.1)	21 (10.1)	0.90 (0.49–1.67)	0.74
Hemicraniectomy	13 (3.4)	6 (3.4)	7 (3.4)	1.01 (0.35–2.96)	0.98
Clot migration	39 (10.2)	16 (9.1)	23 (11.1)	0.82 (0.45–1.51)	0.53
Distal occlusions present at procedure end	31 (8.1)	14 (8.0)	17 (8.2)	0.97 (0.49–1.92)	0.94
Arterial perforation	3 (0.8)	3 (1.7)	0	NA	NA
Arterial dissection	4 (1.0)	0	4 (1.9)	NA	NA
Puncture access complications	12 (3.1)	7 (4.0)	5 (2.4)	1.66 (0.54–5.13)	0.38

### Subgroup analyses

**Figure 3:**
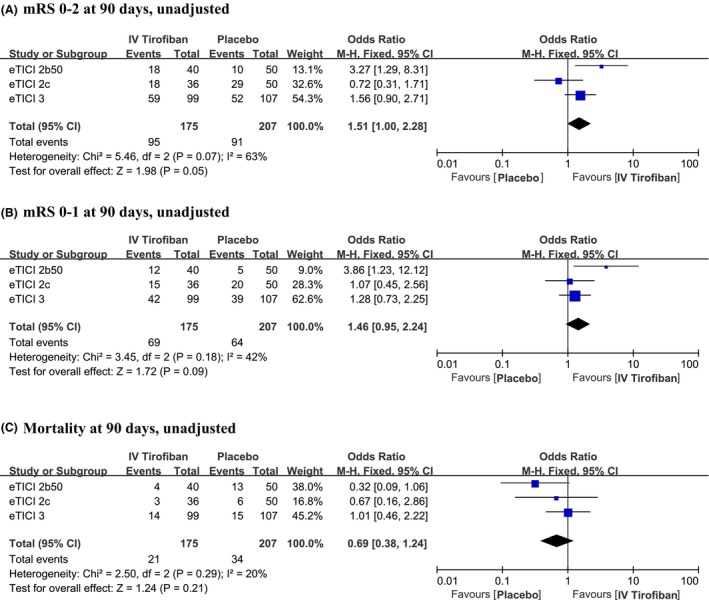
Heterogeneity analysis for intravenous tirofiban effect on outcomes with stratified reperfusion grades.

The effects of tirofiban stratified by reperfusion grade on the primary outcome, the secondary efficacy outcomes, and primary safety outcomes were shown in Figure 3. For the primary functional independence (mRS 0–2) outcome, in unadjusted analysis there was evidence of heterogeneity of treatment effect with different reperfusion levels, *I* ^2^ = 63%, P (interaction) = 0.07. Among patients with eTICI 2b50, the proportion achieving independent functional outcome (mRS of 0 to 2) was increased with tirofiban (45% vs. 20%; OR 3.27; 95% CI, 1.29–8.31).

**Figure 4:**
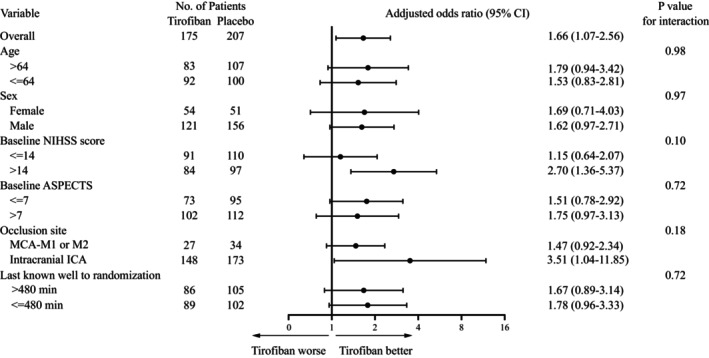
Subgroup analysis.

There was no significant heterogeneity of effect of the functional outcome across the subgroup: age, sex, baseline ASPECTS, occlusion site, time from last known well to randomization (Fig. 4). A trend was noted to magnified benefit in patients with higher NIHSS scores.

## Discussion

This study focused on the population of patients with acute ischemic stroke due to intracranial large artery atherosclerotic disease, which is a main cause of stroke in Asian patients and a determinant of poor outcome. Randomized studies evaluating the outcome of EVT in patients with intracranial LAA were sparse. Among intracranial LAA patients with successful reperfusion (eTICI 2b50‐3), periprocedural intravenous tirofiban treatment was associated with improved functional outcome at 90 days, and rates of death and sICH were comparable between the two groups. The magnitude of treatment effect was sizable, with the number needed to treat for one more functional independent outcome at 90 days of 9.7. Due to the post hoc secondary analysis design and limited size of successful population, all of the results were exploratory.

In the overall RESCUE BT trial, mechanisms of qualifying ischemic stroke were LAA in 45.9%, cardioembolic in 42.8%, other in 2.3%, and unknown in 8.1% and tirofiban was associated with improved outcomes only in the LAA patients.^10^ This finding accords with the pharmacophysiological effects of tirofiban. Tirofiban, a glycoprotein IIb/IIIa receptor inhibitor, exerts a powerful antiplatelet effect by antagonizing the final common step in the aggregation of activated platelets.^16^ Atherosclerotic lesions with their irregular surfaces and disruption of laminar flow streams are particularly likely to activate platelets. This inherent tendency is likely further magnified by mild endothelial injury and plaque disruption caused by EVT device passes during the procedure. Our results are consistent with a beneficial effect of tirofiban in blocking activated platelets, inhibiting acute thrombosis formation, reducing reocclusion at the treatment site, reducing potential artery to artery from the treatment site, and enhancing the dissolution of thrombi distal to the treatment site. In patients with non‐LAA stroke, it seemed that tirofiban could not improve the clinical outcomes. On the contrary, higher proportion of sICH and mortality at 90 days were observed in these patients treated with intravenous tirofiban. Previous study illustrated that low‐dose tirofiban was not associated with sICH in cardioembolic stroke patients treated with EVT.^17^ A subgroup analysis of the RESCUE BT found that tirofiban increased the risk of sICH after EVT in cardioembolized stroke patients.^18^ Therefore, it is still unclear whether intravenous tirofiban is safe or not in patients with non‐LAA stroke, future trials are needed.

Intriguingly, among LAA patients there was evidence of heterogeneity of treatment effect across different eTICI reperfusion levels. Tirofiban was associated with improved outcomes among patients with substantial reperfusion (eTICI 2b50), but no statistically significant effect was seen for patients with excellent (eTICI 2c) or complete (eTICI 3) reperfusion. This observation supports a role for tirofiban in resolving or stabilizing residual microcirculatory obstructions angiographically visible in distal fields at the end of the thrombectomy procedure. Conversely, this finding suggests that an effect of tirofiban upon microcirculatory reperfusion is less marked or absent.

Among LAA patients with successful reperfusion, intravenous tirofiban was associated with an increased rate of any radiologic intracranial hemorrhage but not symptomatic intracranial hemorrhage or mortality. This finding suggests that the antiplatelet effect of intravenous tirofiban may increase rates of any hemorrhagic transformation of bland infarcts but may not predispose to major hemorrhage to the same degree. In addition, the extended EVT treatment time window may have contributed to increased rates of intracranial hemorrhage in both treatment groups in the current study.

The strengths of our study included the large‐scale, double‐blind, and placebo‐controlled design. This study also has several limitations. First, it was a post hoc analysis, multiple testing may increase the risk of Type I errors. All the results should be interpreted with caution. Second, only Chinese patients with LAA stroke were enrolled in the trial, which may reduce the generalizability of study findings. Third, the selection for patients in the extended therapeutic window was based on ASPECTS score according to non‐contrast CT scan rather than CT perfusion assessment, as automated CT perfusion analysis software was not available in many of participating hospitals due to high cost. Fourth, end‐of‐procedure reperfusion grade is an early post‐randomizing rather than baseline patient characteristics, necessitating caution regarding subgroup findings. However, study treatment on average was started coincident with, not before, arterial puncture and only a small fraction of treatment agent was administered before reperfusion grade was assessed. Fifth, analyses are generally more statistically powerful when continuous measures are used rather than dichotomies in the subgroup analysis. Interpretation of the results should be more cautious.

## Conclusions

Among patients with large vessel occlusion due to large artery atherosclerotic stroke and successful reperfusion after endovascular thrombectomy, adjunct intravenous tirofiban was associated with a higher rate or independent functional outcome, without higher rates of symptomatic intracranial hemorrhage or mortality. Confirmatory randomized trials in these patient populations are desirable.

## Author Contributions

Dr Wenjie Zi had full access to all of the data in the study and take responsibility for the integrity of the data and the accuracy of the data analysis. *Concept and design*: Jiacheng Huang, Weilin Kong, Chang Liu, Jiaxing Song, Fengli Li, Saver, Wenjie Zi. *Acquisition, analysis, or interpretation of data*: Jiacheng Huang, Weilin Kong, Chang Liu, Jiaxing Song, Jie Yang, Chengsong Yue, Linyu Li, Jinrong Hu, Yan Tian, Zhouzhou Peng, Changwei Guo, Dahong Yang, Xiang Liu, Jian Miao, Xiao Zhang, Fengli Li, Jeffrey L. Saver, Wenjie Zi. *Drafting of the manuscript*: Jiacheng Huang, Weilin Kong, Chang Liu, Jiaxing Song, Fengli Li, Saver, Wenjie Zi. *Critical revision of the manuscript for important intellectual content*: Jiacheng Huang, Saver, Wenjie Zi. *Statistical analysis*: Jiacheng Huang, Saver. *Administrative, technical, or material support*: Jiacheng Huang, Saver, Wenjie Zi. *Supervision*: Wenjie Zi.

## Funding Information

This study was funded by National Natural Science Foundation of China (No.82071323).

## Conflict of Interest

Dr Saver reported receiving contracted hourly payments for service on clinical trial steering committees advising on rigorous trial design and conduct from Medtronic, Cerenovus, NeuroVasc, Boehringer Ingelheim (prevention only);stock options for service on Clinical Trial Steering Committees advising on rigorous trail design and conduct from Rapid Medical; and contracted hourly payments for service on data safety monitoring committee advising on rigorous trial design, safety, and conduct from MIVI outside the submitted work.

## Patient Consent for Publication

Not applicable.

## Ethics Approval

This study was approved by the ethics of the Xinqiao Hospital, Army Medical University and all participating centers. Written informed consent was obtained from all the patients or their legally authorized representatives.

## Supporting information


**Table S1**.

## Data Availability

Data are available upon reasonable request.
