# Supplementary material for: Intravenous tirofiban following successful reperfusion in intracranial large artery atherosclerotic stroke: A secondary analysis of a randomized clinical trial
Source: Ann Clin Transl Neurol. 2023 Aug 30;10(11):2043–52. doi: 10.1002/acn3.51891 (PMC10646994; doi:10.1002/acn3.51891)
Supplement: Supplementary file 1 — Table S1. [file ACN3-10-2043-s001.docx]

Table S1. Efficacy and safety outcomes in patients due to non-LAA stroke.

| Outcomes | All  (N = 484) | Tirofiban  (N = 252) | Placebo  (N = 232) | Adjusted OR  (95% CI)§ | *P* Value |
| --- | --- | --- | --- | --- | --- |
|  |  |  |  |  |  |
| **Primary efficacy outcome** |  |  |  |  |  |
| Modified Rankin Scale score of 0-2  at 90 days, No./total No. (%) | 249 (51.4) | 128 (50.8) | 121 (52.2) | 0.92 (0.62-1.36) | 0.67 |
| **Secondary efficacy outcome** |  |  |  |  |  |
| Modified Rankin Scale score at 90 days, median (IQR)ǁ | 3 (1-4) | 2 (1-4) | 3 (1-4) | 1.14 (0.83-1.57) | 0.42 |
| Modified Rankin Scale score of 0-1  at 90 days, No./total No. (%) | 185 (38.2) | 97 (38.5) | 88 (37.9) | 0.97 (0.65-1.45) | 0.87 |
| Modified Rankin Scale score of 0-3  at 90 days, No./total No. (%) | 311 (64.3) | 159 (63.1) | 152 (65.5) | 0.88 (0.58-1.34) | 0.56 |
| NIHSS score changes, median (IQR) |  |  |  |  |  |
| Change from baseline to 24 hours* | -4 (-5–2) | -1 (-5–2) | -2 (-5–3) | 0.45 (-1.13–2.03) | 0.58 |
| Change from baseline to 5-7 days  or early discharge* | -4 (-8–1) | -4 (-8–0) | -4 (-9–1) | 1.32 (-0.69–3.34) | 0.20 |
| EQ-5D-5L score at 90 days, median (IQR)* | 0.71 (0.22-0.96) | 0.78 (0.27-0.96) | 0.68 (0.12-0.96) | -0.02 (-0.09–0.04) | 0.47 |
| Substantial reperfusion eTICI score,  No./total No. (%)ǁ |  |  |  | 1.14 (0.80-1.63) | 0.47 |
| 2b | 143 (29.5) | 74 (29.4) | 69 (29.7) |  |  |
| 2c | 53 (11.0) | 23 (9.1) | 30 (12.9) |  |  |
| 3 | 288 (59.5) | 155 (61.5) | 133 (57.3) |  |  |
| Rescue Drug use, No./total No. (%) | 11 (2.3) | 7 (2.8) | 4 (1.7) | 1.94 (0.52-7.22) | 0.33 |
| **Primary safety outcomes** |  |  |  |  |  |
| Symptomatic intracranial hemorrhage | 40 (8.3) | 28 (11.1) | 12 (5.2) | 2.21 (1.07-4.56) | 0.03 |
| Any radiologic intracranial hemorrhage | 167 (34.5) | 96 (38.1) | 71 (30.6) | 1.37 (0.93-2.03) | 0.11 |
| Mortality at 90 days | 83 (17.1) | 52 (20.6) | 31 (13.4) | 1.75 (1.05-2.91) | 0.03 |

Abbreviations: NIHSS, National Institutes of Health Stroke Scale; eTICI, expanded Thrombolysis In Cerebral Infarction grade; EQ-5D-5L, European Quality of Life 5-Dimension 5-level scale; DSA, digital subtraction angiography; OR, odds ratio; CI, confidence interval; NA: not applicable; IQR, interquartile range.

§ Values were adjusted for age, baseline NIHSS score, baseline ASPECTS, occlusion site, and time from last known well to randomization.

ǁ Values were calculated using ordinal logistic model.

* Values were calculated using linear regression model
